# Delayed bowel perforation after instilling over warmed peritoneal dialysate

**DOI:** 10.1186/s12882-021-02343-9

**Published:** 2021-04-20

**Authors:** Xueli Lai, Mingming Nie, Xiaodong Xu, Yuanjie Chen, Zhiyong Guo

**Affiliations:** 1 Department of Nephrology, Shanghai Changhai Hospital, 168 Changhai Road, Shanghai, 200433 P.R. China; 2 Department of Gastroenterology, Shanghai Changhai Hospital, 168 Changhai Road, Shanghai, 200433 P.R. China; 3 Department of Colorectal Surgery, Shanghai Changhai Hospital, 168 Changhai Road, Shanghai, 200433 P.R. China; 4 Intensive Care Unit, Shanghai Changhai Hospital, 168 Changhai Road, Shanghai, 200433 P.R. China

**Keywords:** Peritoneal dialysis, Perforation, Thermal damage, Case report

## Abstract

**Background:**

Peritoneal dialysis (PD) is a safe and home-based treatment for end-stage renal disease (ESRD) patients. The direct thermal damage of abdominal organs is very rare.

**Case presentation:**

We report a peritoneal dialysis patient presented abdominal pain and feculent effluent 3 weeks after he instilled hot dialysis solution. In spite of emergency exploratory laparotomy and active treatment, the patient died of septic shock. Biopsy revealed necrosis and perforation of the intestines.

**Conclusions:**

Delayed bowel perforation by hot fluid is very rare. Standardized performance is of the first importance for peritoneal dialysis patients.

## Background

Peritoneal dialysis (PD) is one of the important kidney replacement treatments of end-stage renal disease patients, which is a safe、convenient and home-based treatment. Patient education is crucial, because any non-standard operating steps may result in various complications. Bowel perforation is an uncommon but serious complication of PD. We report a case of delayed perforation of the small bowel after instilling hot peritoneal fluid. To our knowledge, there have been no previous reports of thermal bowel damage caused by dialysis solution.

## Case presentation

A 63-year-old man was admitted to our hospital complaining that he presented abdominal pain and feculent effluent. He was hospitalized for uremia treated with peritoneal dialysis 5 years ago. The possible reason for uremia was chronic glomerulonephropathy, and the renal biopsy was not performed. He remained on continuous ambulatory peritoneal dialysis without complications for 5 years till he instilled hot dialysis solution, which was heated in a microwave oven on high for 5 min by his new caretaker in a nursing home 3 weeks before. (The caretaker was a fresh man and came from a nursing home without enough education and training of peritoneal dialysis.)The patient went to the emergency room immediately because of severe abdominal pain. There was no signs of bowel perforation and peritonitis. No abnormality was found in abdominal X ray and complete blood count. The peritoneal fluid culture was sent. He was asked to fast for 5 days and administered with preventive antibacterial treatment. Meanwhile he ceased peritoneal dialysis and was converted to non-heparin hemodialysis. The abdominal pain relieved and the peritoneal drainage was clear, so he asked for being discharged from the hospital on the second day against medical advice. After 3 days, the microbiological analysis of the peritoneal fluid revealed growth of *Enterococcus faecium*. He was treated with intravenous (IV) fluids and antibiotics in the emergency room of another hospital. After 10 days of IV treatment, he felt pain in the abdomen again and feculent effluent was found in the peritoneal catheter. He was readmitted to our hospital.

**Fig. 1:**
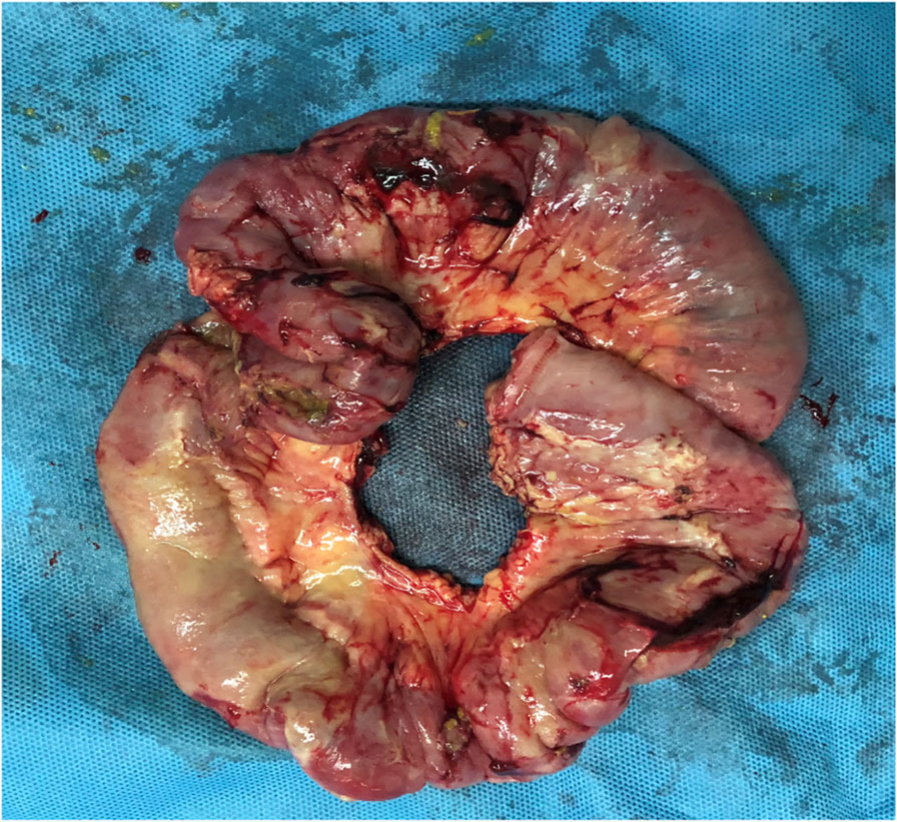
Gross view of the necrotic small bowel: 33 cm of which the largest perforation hole was in the middle

**Fig. 2:**
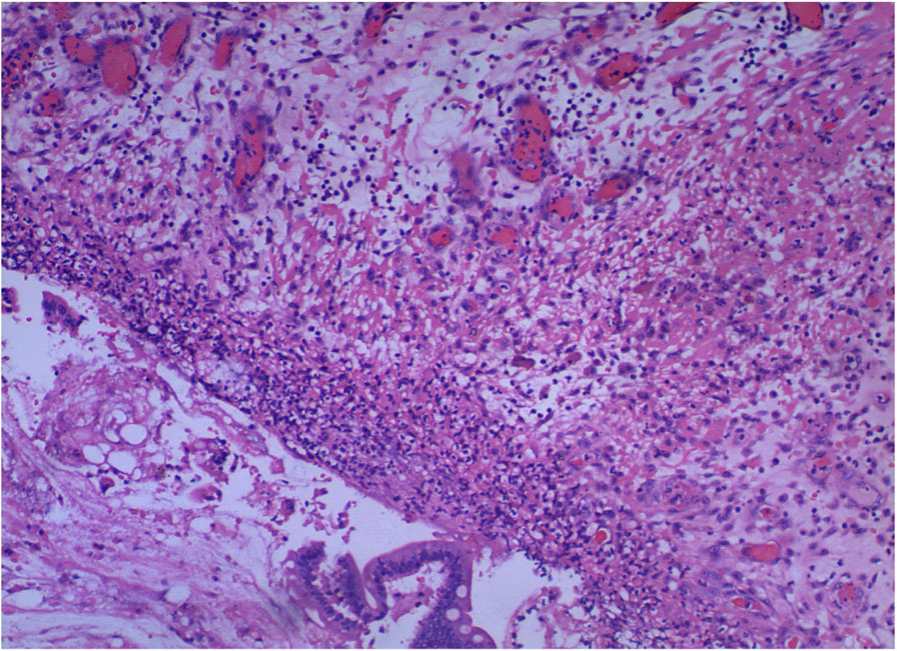
Histologic findings of the small bowel showing hemorrhagic necrosis and neutrophil infiltration of the full-thickness bowel around the largest hole. [hematoxylin and eosin (H&E) stain; original magnification× 100]

Examination on admission indicated that he was afebrile and had a soft、tender abdomen. Blood examination showed white blood cell count 21.88 × 10^9^/l, hemoglobin 72 g/l, neutrophil count 19.89 × 10^9^/l. Abdominal computed tomography (CT) showed peri-hepatic pneumatosis and effusion. No free air or dilated loops of bowel were seen. The patient underwent emergency exploratory laparotomy. A great mount of pus mosses and fecal fluid were found in the abdominal cavity and between intestines. There were multiple perforation holes on the small bowel (the largest one 4*3 cm) and a single perforation (1*1 cm) on the anterior rectum wall. A section of the small bowel was resected (Figs. 1 and 2). Other perforation holes were repaired and ileostomy was performed. The peritoneal catheter was removed. The peritoneal cavity was lavaged with copious amounts of saline solution. The patient developed intestinal fistula after the surgery. Parenteral nutrition, anti-infection treatment and continuous renal replacement therapy (CRRT) were applied. The patient died of septic shock 15 days later.

## Discussion and conclusion

Delayed bowel perforation is an uncommon complication of PD [1]. The peritoneal catheter is believed to be a major risk factor due to its ability to cause erosion or perforation of hollow viscera [2]. To our knowledge, there have been no reports of bowel perforation caused by hot dialysate nor reports of organ damage due to direct exposure to hot dialysate or hot water.

In fact, the small intestinal mucosa is very susceptible to warm ischemia even after short duration. Gastrointestinal perforation, abscess formation and sepsis resulting from thermal injury were reported in percutaneous radiofrequency ablation (PRFA) of hepatic tumor [3] and renal tumor [4]. In another case, a substantial amount of heat created by electrosurgical instruments during surgical operations has been shown to spread throughout the tissue, leading to unintended thermal damage [5].

The development of tissue damage depends on both temperature and the duration of heat exposure [6]. It is believed that increasing the temperature of the tissue to > 45^。^C may lead to irreversible tissue change [7]. To explore the temperature of the dialysate the patient instilled, we repeated the heating course and found that the temperature of peritoneal dialysis solution after heating was up to 80^。^C. Although there were none typical clinical manifestations of bowel perforation during the few post-instilling days, we believe that the thermal injury was lasting and deteriorated, resulting in delayed perforation.

The degree of intestinal tissue injury was evaluated using a grading scale from 0 to 8. Grade 0 is defined as normal mucosa. In grade 1 to 3, the subepithelial space of the villi is increasing. Grade 4 is characterized by denuded of the villi, and grade 5 by loss of the villi. The intestinal crypt layer is injured in grade 6, and the entire intestinal mucosa is necrotic in grade 7 [8]. Grade 8 symbolizes transmural infarction. Histological examination of a surgical specimen in our case revealed full-thickness injury including coagulation necrosis and hemorrhage congestion.

In this case, the immediate surgery and early effective anti-infective therapy might be helpful once the peritoneal fluid culture was positive. However, the diagnosis was delayed rendering treatment difficult and complicated because of the delayed perforation. On the other hand, we are greatly convinced that prevention is better than cure for cases like this. No matter how much emphasis we put on the importance of patient education, it cannot be overstated.

At the meantime, we took measures to prevent such incident from happening again. Firstly, our staff called to each PD patient and his/her relative or caretaker to emphasize that peritoneal dialysate must not be warmed by any other devices except a fixed warmer, which is the essential part of PD training. Secondly, for new patients on PD, we extend the training period and make sure they perform peritoneal dialysis repeatedly and correctly. For outpatients and inpatients, we reinforce reeducation and retraining on peritoneal dialysis as much as possible. Thirdly, we contacted with PD solution Dianeal manufacturers and suggest they should highlight the caution of correct heating procedure on the PD bag. Meanwhile, we call for a sound old-age security mechanism, in which caretakers in the nursing home should be trained with professional knowledge and skills for before they need to take care of the elderly suffering from special diseases.

In conclusion, delayed bowel perforation by overly warm fluid is very rare. Standardized performance is of the first importance for peritoneal dialysis patients. Repeated propaganda and education for PD patients is needed.

## Data Availability

If required, the relevant material can be provided by corresponding author on reasonable request.

## Abbreviations

PD: Peritoneal dialysis
ESRD: End-stage renal disease
IV: Intravenous
CT: Computed tomography
CRRT: Continuous renal replacement therapy
PRFA: Percutaneous radiofrequency ablation
